# Sleep Disorders and Constipation in Autistic Children and Youth: Who Receives Standard of Care Drug Treatments?

**DOI:** 10.1007/s10803-025-06762-7

**Published:** 2025-03-15

**Authors:** Amber M. Angell, Choo Phei Wee, Alexis Deavenport-Saman, Camille Parchment, Chen Bai, Olga Solomon, Larry Yin

**Affiliations:** 1 University of Southern California, Los Angeles, CA USA; 2 University of Florida, Gainesville, FL USA; 3 Children’s Hospital Los Angeles, Los Angeles, CA USA

**Keywords:** Autism, Sleep disorder, Constipation, Co-occurring condition, Healthcare claims

## Abstract

**Purpose:**

The purpose of this retrospective cohort analysis was to investigate sex differences in receipt of standard of care sleep and constipation drug treatments among autistic children and youth with sleep disorder and constipation, respectively.

**Methods:**

We used the data from the OneFlorida + Data Trust to analyze healthcare claims for 19,877 autistic patients with sleep disorder and 32,355 patients with constipation, ages 1 to 22. We used logistic regression to examine sex differences in receiving sleep and constipation treatments, and a multivariate logistic regression model to further assess sex differences in ever receiving sleep and constipation treatments, adjusting for age, race, ethnicity, and urbanicity.

**Results:**

In our multivariate analysis, autistic girls with sleep disorder were 1.27 times more likely than boys to receive sleep treatment (*p* < 0.0001). Although autistic girls with constipation appeared to be 1.10 times more likely than boys to receive treatment, it was not significantly different after adjusting for demographic and socio-economic characteristics (*p* = 0.372). Older children were 1.09 times more likely than younger children to receive sleep treatment (*p* < 0.0001) and 1.07 times more likely to receive constipation treatment (*p* < 0.0001).

**Conclusion:**

We did not find sex differences among autistic children for treatment of constipation, but autistic girls with sleep disorder were significantly more likely to have ever received treatment, which could indicate that girls experience more significant sleep disorders.

**Supplementary Information:**

The online version contains supplementary material available at 10.1007/s10803-025-06762-7.

## Introduction

As autism prevalence reaches 1 in 36 children (Maenner et al., 2023), routine pediatric healthcare practice increasingly involves the management of medical conditions that highly co-occur with autism (Micai et al., 2023). Sleep disorders and constipation are among the most common and can significantly impact children’s wellbeing, behavior, and participation (Díaz-Román et al., 2018; Mazurek et al., 2019; McElhanon et al., 2014). Existing research, however, lacks consistency in how symptoms are defined and measured, as much of it relies on surveys and caregiver report (Klukowski et al., 2015).

Research using real-world clinical data can ameliorate some of these challenges and reflect how these conditions are diagnosed and managed in everyday clinical practice. Such research, however, has shown racial and ethnic (Angell et al., 2022; Broder-Fingert et al., 2013) and sex (Angell et al., 2021, 2022) disparities in rates of co-occurring conditions and in utilization of healthcare services to treat them. There is a need for further research to investigate whether disparities also exist in how these conditions are managed in clinical practice, i.e., whether children receive standard of care drug treatments.

We aimed to address these gaps. In this brief report, we build from our prior work showing that autistic girls had significantly higher rates of sleep and GI disorders than autistic boys (Angell et al., 2021). As in our prior research, we used the same Florida healthcare claims dataset to investigate sex differences in receipt of standard of care drug treatments for sleep and constipation. Based on previous research showing sex disparities in healthcare utilization (Angell et al., 2022; Tint et al., 2017; Xu et al., 2019), we hypothesized that autistic girls with sleep disorders or constipation would be less likely than autistic boys to have corresponding treatment. These preliminary findings examining disparities in receipt of standard of care treatments are intended to contribute to the emerging body of “sex and gender based medicine” (Werbinski et al., 2019) in pediatric healthcare, by providing a starting point for investigating whether sex-based differences in adult health and healthcare outcomes are present in pediatric healthcare.

## Methods

In this retrospective cohort study using Florida administrative healthcare claims data, we asked: Among autistic children and youth with identified sleep disorder or constipation, were there sex differences in receipt of corresponding standard of care drug treatments?

### Data Source and Participants

We received data from the OneFlorida+^1^ Data Trust, a Patient Centered Outcomes Research Institute (PCORI) clinical data research network (PCORnet) that provides patient-level electronic health record (EHR) and administrative claims data (including Medicaid) for most of Florida, transformed to the PCORI Common Data Model. The study was approved as exempt by the University of Florida and University of Southern California Institutional Review Boards.

We received healthcare claims data for children and youth age 21 and younger at the time of their first claim with an autism spectrum disorder diagnosis (ICD-9 200.0, 299.1, 299.8, 299.9; ICD-10 F84.0, F84.5, F84.8, F84.9). Data included healthcare claims from January 2, 2012 to June 30, 2019. We eliminated data from 2019 because the half-year was not comparable to other full years. We sampled patients who had at least one inpatient or long term care claim, or two outpatient claims, with a primary autism diagnosis code in a calendar year (Burke et al., 2014). Because autism can be diagnosed reliably at age 18 months (Hyman et al., 2020), we further limited our sample to children who were at least one-year-old at their first claim with an autism diagnosis. Finally, our sample was limited to patients with any claim with a sleep disorder or constipation diagnosis. This resulted in *N* = 19,877 patients with sleep disorder and *N* = 32,355 patients with constipation, ages 1 to 22.

### Measures

The outcome variables were ever receiving sleep or constipation treatments (yes/no) that corresponded to a sleep disorder or constipation diagnosis, respectively. We grouped ICD-9 and ICD-10 diagnosis codes into clinically meaningful categories using the Healthcare Cost and Utilization Project Multilevel Clinical Classification Software (CCS). Sleep disorders included CCS Level 1 *18* (ICD-9) and Category *259* (ICD-10). Constipation included CCS Level 3 *9.12.1* (ICD-9) and Category *155* (ICD-10; K5900-K5909 only). We used the Federal Drug Agency (FDA) National Drug Code (NDC), and consultation with our clinical experts, to identify both prescription and over the counter (OTC) drugs to treat sleep disorders and constipation. We included OTC drugs because Medicaid recipients (most of our sample) qualify for their coverage.

The predictor variable was sex (male/female). We used age at first claim with an autism diagnosis. We used Rural-Urban Continuum Codes (RUCCs) for urban/rural categorization (U.S. Department of Agriculture, 2013). We categorized patients who used *any* Medicaid as having public insurance; we categorized those who had *only* private insurance claims as having private insurance. We used CCS categories for co-occurring conditions: Anxiety, mood disorder, epilepsy, metabolic disorders, attention deficit hyperactivity disorder (ADHD), and intellectual disorder. We also separated developmental disorder from intellectual disorder to create a separate category.

### Analysis

We described patient demographics and characteristics for receiving any versus no sleep and constipation treatments, using median and interquartile range for continuous variables (age), and frequency and percentages for categorical variables (see Table 1). We used chi-square and Fisher’s exact tests to assess differences in the distribution of patient characteristics, and a Wilcoxon rank-sum test to assess differences in age.

**Table 1:** Characteristics of Florida autistic children and youth with sleep disorder or constipation who received any versus no corresponding treatment, 2012 to 2018

	Patients with sleep disorder (*N* = 19,877)	Any sleep treatment received (*n* = 5,626)	No sleep treatment received (*n* = 14,251)	Unadjusted OR (95% CI)	Difference between groups *P*	Patients with constipation (*N* = 32,355)	Any constipation treatment received (*n* = 1,028)	No constipation treatment received (*n* = 31,327)	Unadjusted OR (95% CI)	Difference between groups *P*
Age (in year), median (IQR) [min, max]	7 (4–11) [1–22]	9 (5–14) [1–22]	6 (4–10) [1–22]	1.10 (1.09–1.10)	**< 0.0001**	6 (3–10) [1–22]	8 (5–13) [1–22]	6 (3–10) [1–22]	1.07 (1.06–1.08)	**< 0.0001**
Sex, n (%)					**< 0.0001**					0.083
Male	15,154 (76.24)	4,080 (72.52)	11,074 (77.71)	1.00		24,561 (75.91)	757 (73.64)	23,804 (75.99)	1.00	
Female	4,723 (23.76)	1,546 (27.48)	3,177 (22.29)	1.32 (1.23–1.42)		7,794 (24.09)	271 (26.36)	7,523 (24.01)	1.13 (0.98–1.30)	
Race, n (%)					0.261					0.041
White	7,096 (35.70)	2,024 (35.98)	5,072 (35.59)	1.00		11,003 (34.01)	349 (33.95)	10,654 (34.01)	1.00	
Black	1,931 (9.71)	587 (10.43)	1,344 (9.43)	1.09 (0.98–1.22)		2,951 (9.12)	118 (11.58)	2,832 (9.04)	1.28 (1.04–1.59)	
Asian	125 (0.63)	31 (0.55)	94 (0.66)	0.83 (0.55–1.24)		231 (0.71)	4 (0.39)	227 (0.72)	0.54 (0.20–1.45)	
Others	164 (0.83)	51 (0.91)	113 (0.79)	1.13 (0.81–1.58)		267 (0.83)	12 (1.17)	225 (0.81)	1.44 (0.80–2.59)	
Refuse to answer/unknown	10,561 (53.13)	2,933 (52.13)	7,628 (53.53)			17,903 (55.33)	544 (52.92)	17,359 (55.41)		
Ethnicity, n (%)					**< 0.0001**					**< 0.0001**
Non-Hispanic	7,645 (38.46)	2,243 (39.87)	5,402 (37.91)	1.00		11,256 (34.79)	397 (38.62)	10,859 (34.66)	1.00	
Hispanic	4,092 (20.59)	947 (16.83)	3,145 (22.07)	0.73 (0.66–0.79)		7,991 (24.70)	190 (18.48)	7,801 (24.90)	0.67 (0.56–0.79)	
Unknown	8,140 (40.95)	2,436 (43.30)	5,704 (40.03)			13,108 (40.51)	441 (42.90)	12,667 (40.43)		
Urbanicity, n (%)					0.267					0.483
No	655 (3.30)	175 (3.11)	480 (3.37)	1.00		792 (2.45)	22 (2.14)	770 (2.46)	1.00	
Yes	18,708 (94.12)	5,372 (95.49)	13,336 (93.58)	1.10 (0.93–1.32)		30,816 (95.24)	993 (96.60)	29,823 (95.20)	1.16 (0.76–1.79)	
Unknown	514 (2.59)	79 (1.40)	435 (3.05)			747 (2.31)	13 (1.26)	734 (2.34)		
Insurance, n (%)					NA					NA
Medicaid	18,626 (93.71)	5,626 (100)	13,000 (91.22)	1.00		30,181 (93.28)	1,028 (100)	29,153 (93.06)	1.00	
Private	1,251 (6.29)	0 (0)	1,251 (8.78)	NA		2,174 (6.72)	0 (0)	2,174 (6.94)	NA	
Co-occurring conditions, n (%)										
Constipation	11,214 (56.42)	3,904 (69.39)	7,310 (51.29)	2.15 (2.02–2.30)	**< 0.0001**	NA	NA	NA	NA	NA
Sleep	NA	NA	NA	NA	NA	11,214 (34.66)	554 (53.89)	10,660 (34.03)	2.27 (2.00–2.57)	**< 0.0001**
Anxiety	7,865 (39.57)	2,931 (52.10)	4,934 (34.62)	2.05 (1.93–2.19)	**< 0.0001**	10,243 (31.66)	480 (46.69)	9,763 (31.16)	1.93 (1.71–2.19)	**< 0.0001**
Mood	5,284 (26.58)	2,140 (38.04)	3,144 (22.06)	2.17 (2.03–2.32)	**< 0.0001**	6,513 (20.13)	325 (31.61)	6,188 (19.75)	1.88 (1.64–2.15)	**< 0.0001**
Epilepsy	7,571 (38.09)	3,237 (57.54)	4,334 (30.41)	3.10 (2.91–3.30)	**< 0.0001**	10,290 (31.80)	459 (44.65)	9,831 (31.38)	1.76 (1.56–2.00)	**< 0.0001**
Metabolic	2,089 (10.51)	990 (17.60)	1,099 (7.71)	2.56 (2.33–2.80)	**< 0.0001**	3,040 (9.40)	185 (18.00)	2,855 (9.11)	2.19 (1.86–2.58)	**< 0.0001**
ADHD	14,510 (73.00)	4,299 (76.41)	10,211 (71.65)	1.28 (1.19–1.38)	**< 0.0001**	20,199 (62.43)	743 (72.28)	19,456 (62.11)	1.59 (1.38–1.83)	**< 0.0001**
ID	3,655 (18.39)	1,913 (34.00)	1,742 (12.22)	3.70 (3.43–3.99)	**< 0.0001**	5,062 (15.65)	326 (31.71)	4,736 (15.12)	2.61 (2.28–2.98)	**< 0.0001**
DD	16,010 (80.55)	4,649 (82.63)	11,361 (79.72)	1.21 (1.12–1.31)	**< 0.0001**	26,773 (82.75)	897 (87.26)	25,876 (82.60)	1.44 (1.20–1.74)	**< 0.0001**

ADHD = Attention Deficit Hyperactivity Disorder; ID = Intellectual Disability; DD = Developmental Disorder

We used a logistic regression model to examine sex differences in receiving sleep and constipation treatments. We then used a multivariate logistic regression model to further assess sex differences in ever receiving sleep and constipation treatments, adjusting for age, race, ethnicity, and urbanicity. These results are expressed in odds ratios (OR) with associated 95% confidence intervals, using a 5% significance level with a two-sided test. We also examined the non-additive effects of sex differences on receiving sleep and constipation treatments by anxiety (by including sex and anxiety condition as interaction term) in the multivariate model. We performed all statistical computations in Stata/SE 17.0 (StatCorp, College Station, TX).

## Results

As shown in Table 1, among 19,877 autistic children and youth with sleep disorders, girls with sleep disorders were significantly more likely than boys with sleep disorders to have received any sleep treatment (*p* < 0.0001). There were also significant differences in ever receiving sleep treatment by age (older), ethnicity (non-Hispanic/Latino), and all other reported co-occurring conditions (all *p* < 0.0001). Among 32,355 autistic children and youth with constipation, there was no significant difference between boys and girls in ever receiving treatment (*p* = 0.083). There were significant differences in ever receiving treatment for constipation: Children receiving treatment were more likely to be older, less likely to receive treatment if they were Hispanic/Latino, and more likely to receive treatment if they had other co-occurring conditions (all *p* < 0.0001).

Table 2 is the multivariate logistic regression model that assessed sex differences in ever receiving sleep and constipation treatments, adjusting for demographic and socioeconomic characteristics. According to the multivariate analysis, among autistic children and youth with sleep disorders, girls were 1.27 times more likely to receive sleep treatment compared to boys, after adjusting for demographic and socioeconomic characteristics (95%CI = 1.14 to 1.41, *p* < 0.0001). Older children were 1.09 times more likely than younger children to receive sleep treatment (95%CI = 1.08 to 1.10, *p* < 0.0001). Among autistic children and youth with constipation, although girls appeared to be 1.10 times more likely to receive treatment compared to boys, it was not significantly different after adjusting for demographic and socio-economic characteristics (95%CI = 0.89 to 1.35, *p* = 0.372). Older children were 1.07 times more likely than younger children to receive constipation treatment (95%CI = 1.05–1.08, *p* < 0.0001). We also assessed sex differences on ever receiving sleep and constipation treatments (respectively) by co-occurring anxiety but found no significant differences for either outcome variable (interaction *p* = 0.166 and *p* = 0.088, respectively; see Figs. 1 and 2 in supplemental materials).

**Table 2:** Multivariate logistic regression model assessing sex differences in ever receiving treatment among Florida autistic children and youth with sleep disorders or constipation

Variables	Sleep treatment			Constipation treatment		
	Adjusted OR	95% CI	*P* value	Adjusted OR	95% CI	*P* value
Sex						
Male	1.00	--	--	1.00	--	--
Female	1.27	(1.14–1.41)	**< 0.0001**	1.10	(0.89–1.35)	0.372
Age	1.09	(1.08–1.10)	**< 0.0001**	1.07	(1.05–1.08)	**< 0.0001**
Race						
White	1.00	--	--	1.00	--	--
Black	1.12	(1.00–1.26)	0.555	1.27	(1.02–1.59)	0.033
Asian	0.93	(0.53–1.63)	0.797	0.80	(0.25–2.66)	0.710
Others	1.21	(0.84–1.75)	0.304	1.67	(0.90–3.12)	0.104
Ethnicity						
Non-Hispanic	1.00	--	--	1.00	--	--
Hispanic	1.02	(0.90–1.16)	0.746	0.88	(0.69–1.14)	0.345
Urbanicity						
No	1.00	--	--	1.00	--	--
Yes	1.23	(0.99–1.53)	0.064	1.05	(0.64–1.73)	0.837

## Discussion

In this analysis of administrative healthcare claims, we did not find sex differences among autistic children for treatment of constipation, but autistic girls with sleep disorders in our sample were significantly more likely than autistic boys to have ever received standard of care treatments. Research has consistently found higher rates of sleep disorders among autistic girls (Angell et al., 2021; Estes et al., 2022), that extend into adulthood (Charlton et al., 2023; Jovevska et al., 2020). This mirrors the general population; women are more likely to experience sleep disorders, including insomnia, compared to men (Lok et al., 2024; Mong & Cusmano, 2016; Suh et al., 2018). Higher treatment rates could indicate a sex-based disparity, or it could be that autistic girls experience more significant sleep disorders, as healthcare claims only indicate which children have been diagnosed and do not include information about the severity of the condition. The latter is more likely, given patterns in the general population. In other words, it is more likely that the differences in treatment prescribing are due to more significant sleep problems in girls, i.e., that warrant treatment. More research is needed to determine if there are differences in the type and duration of sleep challenges that girls vs. boys experience, whether various treatments are equally effective for males and females, and the potential role of menstruation and sex hormones in autistic girls, particularly given the age trends in our data (Lok et al., 2024; Mong & Cusmano, 2016).

In our unadjusted model, Hispanic/Latino autistic children with sleep disorders or constipation were less likely than non-Hispanic/Latino children to receive respective standard of care treatments. While this difference was no longer significant after adjusting for other demographic and socio-economic characteristics, an important consideration regarding documented healthcare disparities and medical treatments for sleep and/or constipation is cultural beliefs, values, and preferences. Hispanic/Latino caregivers may be more likely to manage childhood ailments at home and to use complementary health approaches and products purchased in stores selling products from Latin America (Acorda et al., 2020; Angell et al., 2023). A study of prescription practices of gastroenterologists found that OTC medications are the primary recommended treatment for chronic constipation in the general population, and despite feeling that generic treatments are equally effective as store brand treatments, the majority of gastroenterologists recommended name brand medications and underestimated the cost savings of store brands (Menees et al., 2015). More research is needed to determine which products Hispanic/Latino families prefer to treat their autistic children’s constipation; whether provider prescribing practices deter families from purchasing recommended OTC medications; and what other barriers to care may influence our finding. Given the large percentage of missing racial and ethnic data in our sample of Florida Latinos, further research is also needed to determine if racial or ethnic disparities exist across different states, investigating potential differences among various Hispanic/Latino subgroups, as significant variability exists in this ethnic group in the US (Fenelon et al., 2017; Garcia et al., 2018).

Our finding that older children were significantly more likely to receive sleep and constipation treatments is unsurprising, as families are likely to manage symptoms on their own before trying medical treatments (Hutchinson, 2020; Keenan et al., 2007; Lucas et al., 2015; Rooney et al., 2023). This finding may reflect a limitation of our study: Because we aggregated both diagnoses and receipt of treatment at the yearly level, and because this preliminary analysis was not longitudinal, younger children in our dataset who were less likely to receive standard of care treatments may simply not have been diagnosed yet. However, it could also indicate, as previous research shows, that parents wait to try treatment such as melatonin until the sleep problems are persistent over time and/or more severe (Hutchinson, 2020; Keenan et al., 2007; Lucas et al., 2015; Rooney et al., 2023). Our future work will address this limitation by incorporating time into the analysis.

Another limitation of this preliminary study is that not all OTC drugs are listed in patients’ charts. Although our sample, which was 94% Medicaid recipients, can use Medicaid coverage for OTC drugs, this may not be true for all; for example, for patients whose families purchase OTC melatonin or Polyethylene glycol 3350, commonly known as MiraLAX, neither would be reported in our dataset as a filled prescription. Our large Medicaid sample, however, increases the likelihood that OTC drugs are listed in patients’ records.

## Conclusion

In this brief report, we present preliminary findings suggesting potential sex and ethnic disparities in receipt of standard of care treatments for sleep disorder and constipation among autistic children and youth. In doing so, we aim to contribute to the emerging body of literature bringing “sex and gender based medicine” into pediatric healthcare, to investigate potential sex-based differences in health and healthcare outcomes, that may differ in children compared to adults (Werbinski et al., 2019).

## Electronic supplementary material

Below is the link to the electronic supplementary material.

Supplementary Material 1

## Footnotes

1 We use the current name, the OneFlorida + Data Trust, but when we received data it was called the OneFlorida Data Trust. We received only Florida data; it was renamed when it expanded to include parts of Georgia and Alabama.
