# Supplementary material for: Sleep Disorders and Constipation in Autistic Children and Youth: Who Receives Standard of Care Drug Treatments?
Source: J Autism Dev Disord. 2025 Mar 15;55(12):4365–71. doi: 10.1007/s10803-025-06762-7 (PMC12589277; doi:10.1007/s10803-025-06762-7)

**SUPPLEMENTAL MATERIALS**

**Figure 1. Sex difference on receiving sleep treatment by anxiety co-occurring condition by including sex and anxiety condition interaction term in the multivariate model shown in Table 2.**


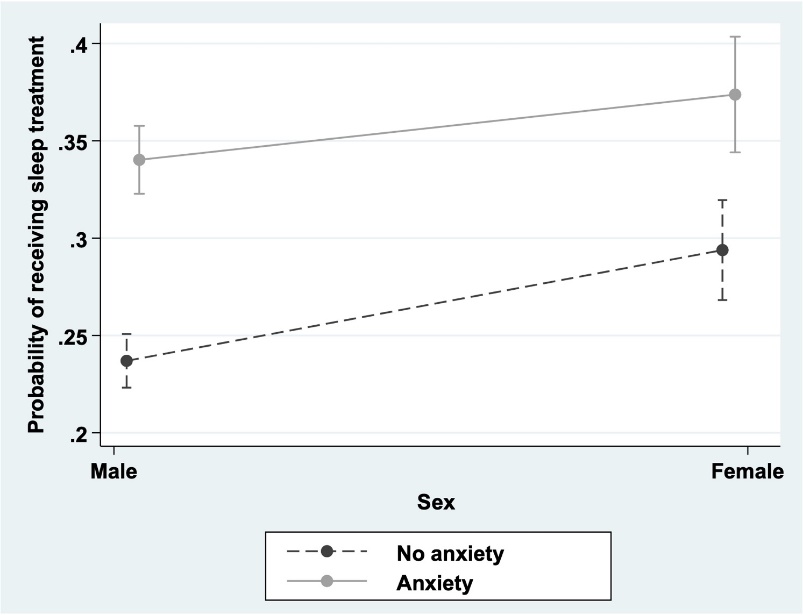


**Figure 2. Sex difference on receiving constipation treatment by anxiety co-occurring condition by including sex and anxiety condition interaction term in the multivariate model shown in Table 2.**


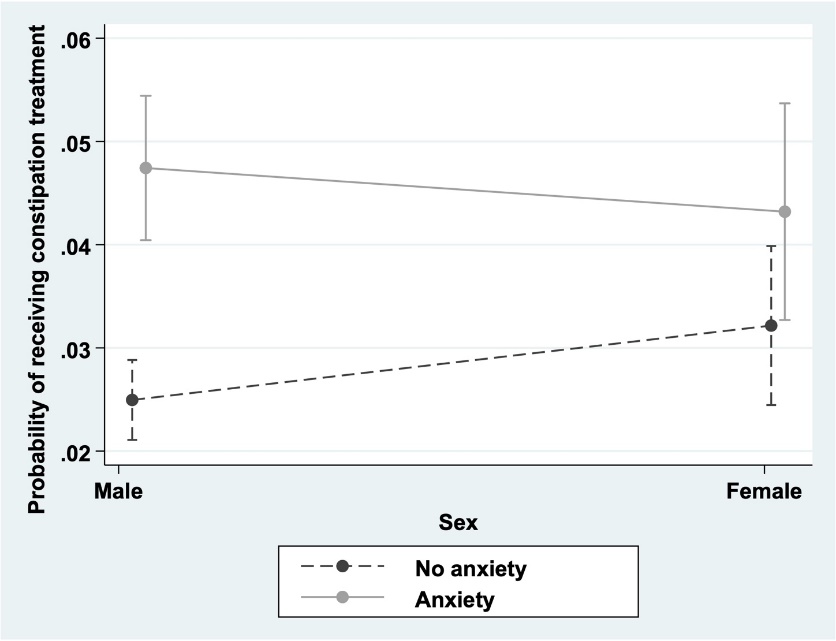

Supplement: Supplementary file 1 — Supplementary Material 1 [file 10803_2025_6762_MOESM1_ESM.docx]
